# Barriers and Facilitators to Using Virtual Reality for Body Image Work in Eating Disorders: A Qualitative Study

**DOI:** 10.1192/bjo.2026.11224

**Published:** 2026-06-30

**Authors:** Juliet Cartwright, Laura Chapman, Lucy Biddle, Lucy Yardley, Helen Bould

**Affiliations:** 1 Bristol Medical School, University of Bristol, Bristol, United Kingdom; 2 School of Psychological Science, University of Bristol, Bristol, United Kingdom; 3 Gloucestershire Health and Care NHS Foundation Trust, Gloucester, United Kingdom

## Abstract

**Aims:**

Many people with eating disorders experience body image disturbance, and there is evidence to suggest that body image difficulties not effectively addressed through eating disorder treatment can contribute to later relapse. Virtual reality (VR) interventions designed to target body image difficulties associated with eating disorders have begun to demonstrate promising evidence of efficacy. However, little is known about the perspectives of people with eating disorders and those who care for them around such interventions. This study aims to explore stakeholders’ views of factors that could impede or support use of such interventions.

**Methods:**

Eleven young people with lived or living experience of eating disorders (PWLE), four parents/carers of people with eating disorders, and five clinicians with experience of treating eating disorders took part in semi-structured interviews or focus groups. Qualitative data was coded inductively and analysed using reflexive thematic analysis. Themes and subthemes were iteratively refined through discussions between the authors.

**Results:**

Five themes were generated. These were *getting the timing right*: highlighting the importance of timing within a person’s treatment journey, with suggestions for optimal timing; *tailoring the intervention to the individual*: all stakeholders agreed that individualisation to the person accessing treatment was critical; *thoughtfully empowering agency*: participants suggested that a degree of control over most aspects of the intervention would be helpful, with some key caveats; *providing the right support*: suggestions were made about both the necessity of support, and what this support might look like; and an overarching theme of *on balance is it worth while?* This overarching theme involved participants carefully weighing up the risks and benefits of using such an intervention. It included three subthemes: *hope of efficacy; fear of difficulty*; and *desperate belief in necessity*.

**Conclusion:**

PWLE, parents and carers and clinicians shared valuable, detailed insights into how they view body image interventions in VR, the key challenging areas and suggestions for how they could be done well. They highlighted that getting these aspects right may make such an intervention on balance worthwhile. Ongoing co-production in order to optimise these components of the intervention should be utilised in the development of VR body image interventions, as they will likely make the difference as to whether people will choose to try them and whether they will be effective.

